# TouchScope: A Passive-Haptic Device to Investigate Tactile Perception Using a Refreshable Braille Display

**DOI:** 10.5334/joc.271

**Published:** 2023-04-14

**Authors:** Ana Baciero, Manuel Perea, Jon Andoni Duñabeitia, Pablo Gómez

**Affiliations:** 1 Bournemouth University, United Kingdom; 2 Universitat de València, Spain; 3 Nebrija University, Spain; 4 The Arctic University of Norway, Norway; 5 C.S.U.S.B., Palm Desert Campus, United States; 6 Skidmore College, NY, USA

**Keywords:** Tactile perception, haptic perception, apparatus, braille

## Abstract

The sense of touch is underrepresented in cognitive psychology research. One of the reasons is that controlling the timing of stimulus presentation, which is a hallmark of cognitive research, is significantly more difficult for tactile stimuli than visual or auditory stimuli. In the present work, we present a system to display tactile stimuli (braille cells) and collect response time with the capability for static and dynamic (passive haptic) stimuli prsentation that will contribute to the development of tactile research. While the system requires some construction, it can be put together with commercially available materials. Here, we present the step-by-step instructions for constructing the tool, the code used to control it, and some basic experiments to validate it. The data from the experiments show that the device can be used for a variety of tactile perception experiments.

Research on perception, attention and reading has been centred on the sense of vision, while tactile/haptic perception is understudied. Note that, in perception research, the term *tactile* is generally used to describe the sensations resulting from cutaneous stimulation, whereas the term *haptic* is used to describe those sensations that involve cutaneous stimulation and kinesthetics (see McGlone & Reilly, 2010; Wolfe et al., 2014).

One of the reasons (among many) for this state in the field is that presenting stimuli to the sense of touch—while accurately controlling their presentation—is significantly more difficult than presenting and controlling visual or auditory stimuli. In the visual modality, at the cost of an inexpensive computer, scientists have access to many platforms to carry out two critical tasks for research: 1) selecting which stimulus is presented and 2) controlling for how long it is presented (e.g., PsychoPy: Peirce et al., 2019). This has allowed scholars to design several paradigms and techniques that carefully control the presentation of visual stimuli. For instance, reading researchers have developed methods such as masked priming (Forster & Davis, 1984), rapid serial visual presentation (Forster, 1970), moving window (McConkie & Rayner, 1975), or the gaze-contingent boundary change technique (Rayner 1975). Such control has allowed researchers to collect high-quality data, avoiding as much noise as possible, adequate for replicating effects and developing sophisticated computational models of word recognition and reading. Similarly, in auditory perception, even older technologies allow for some control over the timing of aural stimuli, like the classic dichotic listening tasks (Broadbent, 1958; Cherry, 1953), or more contemporary techniques like “subliminal” speech priming (Kouider & Dupoux, 2005).

Although some paradigms allow tactile researchers to time the presentation of stimuli, there are no devices equivalent to simple computers, speakers, and monitors used for research in other sensory modalities. Here, we aim to contribute to making tactile research more straightforward by providing researchers with a system to select and present a tactile stimulus among a set of stimuli, with some control over the presentation timing. We use a refreshable braille display placed on a moving (or static) platform. This technology allows us to explore tactile perception phenomena, but its natural application is to study braille reading.

## The Braille Writing System

The braille writing system was invented around 200 years ago by Louis Braille. Different patterns of raised dots in a 2 × 3 matrix, the braille cell, represent the written symbols of language (e.g., a = 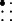). One of the most common ways to access braille written information nowadays is via refreshable braille displays, which are electromechanical devices that allow braille readers to interact with computers, smartphones, and other gadgets (Perkins School for the Blind, International Council on English Braille & Library of Congress, 2013). Braille displays use 8-dot braille cells (i.e., 2 × 4 matrices; e.g., a = 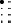), in which the bottom row is used, among other things, to make computer interaction easier or to represent some text characteristics that otherwise would need the use of another cell (e.g., uppercase A = 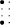; rather than A = 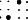 in 6-dot braille cells). The braille writing system offers a great set of stimuli to researchers interested in the sense of touch, both those involved in reading research (e.g., Perea et al., 2012; Veispak et al., 2012) and those interested in tactile and haptic perception in general (see Baciero et al., 2021 for an example of tactile congruency effects, and Heller & Kennedy, 1990, for an example of tactile shape perception and perspective taking).

## Requirements for a tactile stimulus presentation tool

Any device to study tactile and haptic processes requires several features to accurately display and control the stimuli, similar to what researchers in the visual and auditory modalities have; this research aims to fulfill the following requirements:

1. *Stimulus control*. The system has to allow researchers to control stimulus onset, speed of presentation (when involving movement), and trial duration.
2. *Trial customization*. The tool should allow researchers to modify those parameters across trials.
3. *Modality appropriateness*. The tool should allow the display of the stimuli through either friction with the skin or poking a specific location skin—note that these are crucial elements of haptic perception and braille reading.
4. *Automaticity*. The tool needs to present the stimuli in an programmatic manner (as in the experiments in other modalies).
5. *Reproducibility and portability*. Everybody should be able to build it and move it where needed at reasonable costs.

It is important to note that vibration has traditionally been used to study tactile perception, and while our goal was not to implement vibration, the philosophy presented in this work can readily accommodate it.

Advances in technology during approximately the last decade have allowed the creation of fully automated devices that researchers can use to investigate tactile perception in different areas. For instance, researchers on braille reading have designed tools that can automatically track finger movements, thus allowing precise hand movement analyses.^1^ For example, Breidegard et al. (2008) created an *automatic finger tracking system* that videotapes and tracks finger movements from above and below the hand. Hughes (2011) developed a tool to measure the finger’s movements using a digitalizing tablet and attaching a digitalizing pen to the participant’s index fingertip. Aranyanak and Reilly (2012) devised a finger-tracking system using a Wii-remote control that can be used with both paper and refreshable braille displays, and controls in a precise manner finger position within the word/text. Lei et al. (2019) tracked finger position by attaching electromagnetic sensors to the fingernails. Additionally, some refreshable braille displays (e.g., Active Braille; Help Tech., 2018) can track the finger’s position, thus providing information about reading flow and finger movements (see Perea et al., 2015).

While all of these tools are great to analyze the reading movements and provide some stimulus control and customization, they do not offer precise control over the timing of the presentation, as the participant actively moves their hand to perceive the stimuli. Hence, they might not be the ideal tools for experiments with many trials, or those using electrophysiological techniques. The hand and finger movements that individuals make, result in extremely variable response times (see Lei et al., 2019). Furthermore, they may generate motor artifacts in electrophysiological measures, so finding/visualizing the underlying cognitive effects is challenging.

Researchers on texture perception have developed other tools that overcome some of the limitations of finger-tracking using *passive haptic perception* (i.e., the perception mediated by variations in cutaneous stimulation and kinesthesis in which the perceiver does not have control over picking up stimulus information; Loomis & Lederman, 1986). For example, Reales-Avilés et al. (2010) developed a *Tactile Spinning Wheel* where different tactile stimuli fixed onto a rotating circular platform can be presented to the fingertips while controlling for spinning velocity and stimuli position. Oddo et al. (2011) and Moungou et al., (2016) designed robotic devices that slide stimuli against a finger-pad, controlling the sliding speed and force. Richardson et al., (2000) devised a “Tactile Display System” that can both track the finger movements (active) during tactile exploration of different stimuli and guide such finger movements (passive) controlling the speed and position. While these tools have proved to be vey useful in tactile perception research, they also present limitations in terms of the *customizability* and *automaticity* properties we look for in a system; besides, they use either proprietary or fixed hardware that lacks *portability*.

To overcome these limitations, we have developed *TouchScope*, a passive-haptic-perception tool that can help researchers conduct experiments on braille reading, and tactile/haptic perception. This tool fulfills all requirements outlined above: 1) it allows the control of the stimuli presentation timing; 2) researchers can customize trial parameters; 3) it allows the display of stimuli through rubbing on the skin while controlling the hand/arm movements, thus allowing more precise response times and avoiding motor artifacts that may induce noise in cognitive neuroscience techniques; 4) it presents stimuli in an automated manner; and 5) it uses open-source software so it can be recreated in any other laboratory with no extra cost, and has a simple design that makes it portable. We hope that this device will improve our knowledge of the cognitive processes that underlie tactile/haptic phenomena by creating paradigms similar to those used in the visual modality.

## TouchScope. The Device

### Hardware

**Figure 1:**
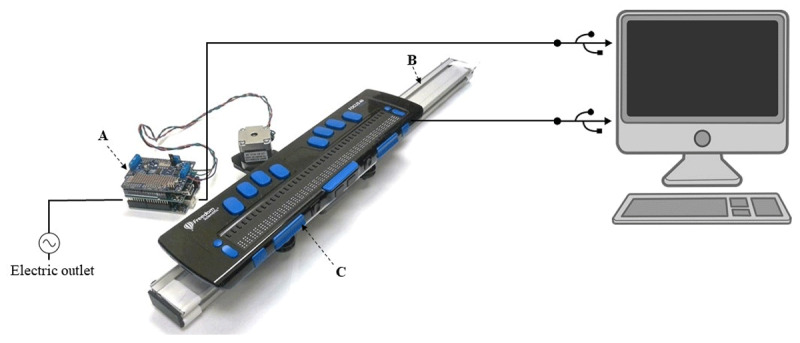
Figure 1 Diagram of the TouchScope tool and its connections. **A)** Arduino Uno board and Adafruit motor shield. **B)** Motorized linear bearing. **C)** Refreshable braille display.

The tool has three main components: 1) a motorized linear bearing; 2) an Arduino Uno board with a motor shield; and 3) a refreshable braille display (see Figure 1). These components are described below one by one.

#### Motorized linear bearing

This is the mechanical part of the system and was created following Dring’s (2013) simple camera slider project (https://www.inventables.com/projects/simple-camera-slider; step-by-step instructions and technical drawings uploaded to our osf project too: https://osf.io/tyzvp/).

**Figure 2:**
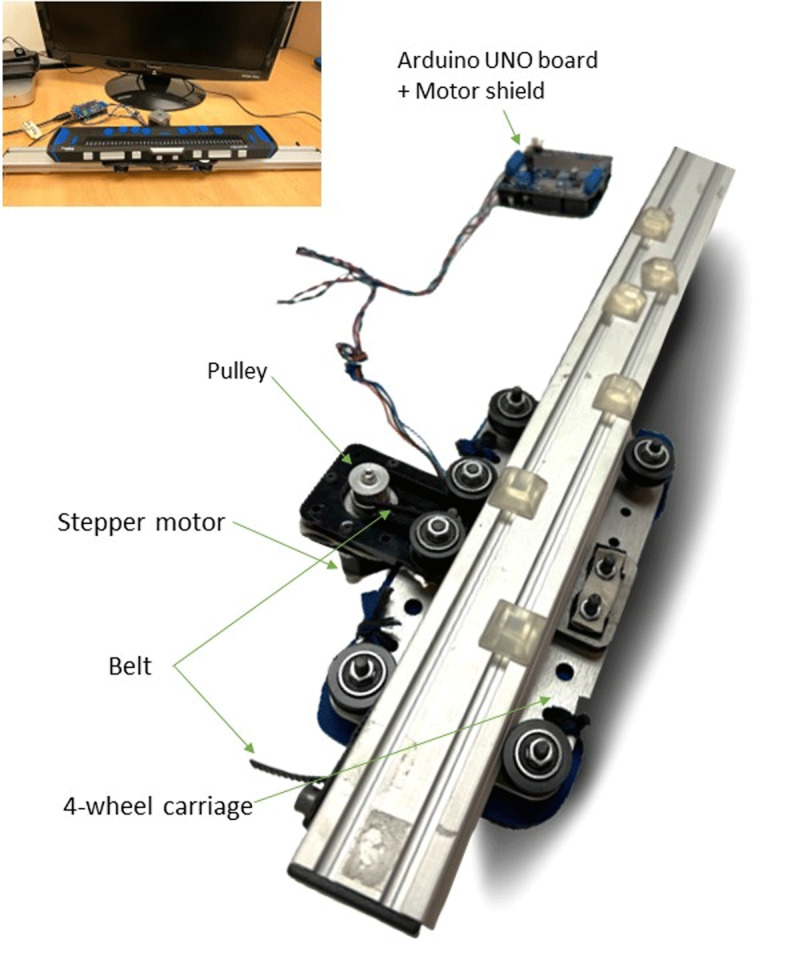
Main components of the motorized linear bearing. The main picture shows the linear bearing upside down while the picture in the top left corner shows the whole system in place (i.e., motorized linear bearing + refreshable braille display on top + circuit).

In a nutshell, we used a NEMA 14 bipolar stepper motor – motor whose full rotation is divided into equal steps – that has 200 steps per revolution and attached to a 160 × 90 mm 4-wheel carriage. This carriage was placed on a rail – aluminium extrusion linear bearing. A 2 mm-pitch toothed belt surrounding the rail was connected to the stepper motor via an 18-tooth pulley, transferring the motor’s motion to the carriage and allowing linear (horizontal) movement (see Figure 2).

#### Circuit

The motor is connected to an Arduino Uno board, a circuit board (i.e., ATmega328-based microcontroller board) that is open-source and contains 14 digital input/output pins plus six analog inputs, a 16-MHz crystal oscillator, a USB connection, a power jack, an In-Circuit Serial Programming header, and a reset button (Arduino.cc., 2020). This board can be powered either from a USB connection or from an AC-to-DC adapter. The board can operate on an external supply of 6 to 20 volts. Each of the 14 digital pins operates at 5 Volts and can be used as an input or output (D’Ausilio, 2011). In addition, an Adafruit Motor Shield is attached to the board to drive the stepper motor, controlling the speed, direction, and distance of the movement.

As shown in Figure 1, the moving platform is connected to the Arduino Uno board through the motor shield (to which the stepper motor is wired); the Arduino Uno board is powered via an AC-to-DC adapter and connected to a MacOS computer through a USB cable. The macOS system was chosen because it has a built-in accessibility feature (VoiceOver) that allows communication with the refreshable braille display.^2^

#### Braille display

On top of the motorized carriage, we placed a refreshable braille display (Focus 40 Blue, Freedom Scientific). This braille display was connected to a computer through a USB cable.

As already mentioned, TouchScope can be used not only to examine braille reading, but also to explore other tactile/haptic perception phenomena that can be examined by taking advantage of the textures that are allowed in braille.

### Software

TouchScope uses three pieces of software: 1) Arduino Integrated Development Environment (IDE); 2) an executable Bash script – a text file containing a sequence of commands that can be executed in the computer’s terminal; and 3) VoiceOver. Both the Arduino IDE and the Bash shell are open-source software, with plenty of information available on-line, and the VoiceOver utility, as stated above, is a tool fully integrated into the macOS X operating system. Hence, there is no extra cost associated with the operation of TouchScope.^3^

#### Arduino IDE

Through the Arduino IDE (see appendix and osf repository for the commented code) we control the carriage speed (in rpm), distance (in steps), and direction of motion (left-right or right-left).

To set up the speed and distance, we first need to calculate our device’s steps per millimeter with the following formula:

\frac{{{\rm{Steps}}\_{\rm{per}}\_{\rm{revolution\ microstepping}}\_{\rm{factor}}}}{{{\rm{Belt}}\_{\rm{pitch\ Pulley}}\_{\rm{teeth}}\_{\rm{number}}}}

As stated above, we used a 200 steps/revolution stepper motor, an 18 teeth pulley, a 2 mm pitch belt, and no microstepping factor – although one could include this if wanted an even more precise movement control. Hence, our TouchScope moves 5.56 steps/mm. That is, 35.9 mm/revolution (i.e., 200 steps per revolution/5.56 steps per millimeter). Of course, these numbers would change if any pieces are modified (e.g., using a 20 teeth pulley, which is more common than the 18 teeth one, the carriage will move 40 mm/rev). Knowing this, we can calculate the rpm (speed) and steps (distance).^4^ For instance, to move the carriage for 50 mm at 40 mm per second we will need:

- 56 steps_per_mm × 50 mm = *278 steps*
-
\frac{{40{\rm{mm}}\_{\rm{per}}\_{\rm{sec}}60{\rm{sec}}\_{\rm{per}}\_{\rm{min}}}}{{35.9{\rm{mm}}\_{\rm{per}}\_{\rm{rev}}}} = 66.9\,rpm

Once these parameters have been set up, the code can be uploaded to the Arduino board’s memory. This only needs to be done once – unless one wants to change the parameters – and then the experimenter just needs to execute the bash script in the experimental session.

#### Bash script

Through the executable Bash script we can: 1) trigger the movement of the platform via communication with the serial monitor of the Arduino IDE; 2) present the trials on the screen via the computer terminal; 3) record responses (e.g., keys pressed, response time) in separate files (see appendix and osf repository for the commented code).

These software elements are synchronized to control the platform’s movement, stimuli presentation, and response recording. Specifically, through the Arduino IDE, we control the carriage speed (in rpm) and distance (in steps); and through the executable Bash script we can: 1) trigger the movement of the platform via communication with the serial monitor of the Arduino IDE; 2) present the trials on the screen via the computer terminal; and 3) record responses (e.g., keys pressed, response time) in separate files.

In addition to the Arduino IDE and the Bash executable, we used the VoiceOver accessibility feature to send the stimuli from the computer terminal to the refreshable braille display. This feature just needs to be enabled in the computer before running the experiment.

## Device Assessment

To offer an idea of the usefulness of TouchScope in the study of haptic/tactile perception, we designed a study that examined the perceived similarity of pairs of braille letters using a same-different task, common in studies of visual letter similarity (e.g., Courrieu et al., 2004), with individuals with no prior knowledge of braille. The specific details are given in the online appendix (https://osf.io/tyzvp/). Here, we report a brief summary of the method and results.

To be able to assess TouchScope’s value, we had two experimental procedures: some participants used the dynamic mode of TouchScope, hence doing the task via passive haptic braille perception (i.e., *passive procedure*), and others used the static mode of TouchScope, thus moving their finger across the refreshable braille display (i.e., *active procedure*). Our main goal was to compare participants’ performance between the two procedures (accuracy and response time variability).

We used all possible 2-letter combinations: 676 pairs, out of which 26 were the same two letters (e.g., 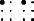), and 650 two different letters (e.g., 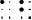). Five lists of pairs were created (130 *same*,130 *different* pairs). Participants were 91 and 86 undergraduate students in the Active- and Passive-procedure, respectively. They were not braille readers.

**Figure 3:**
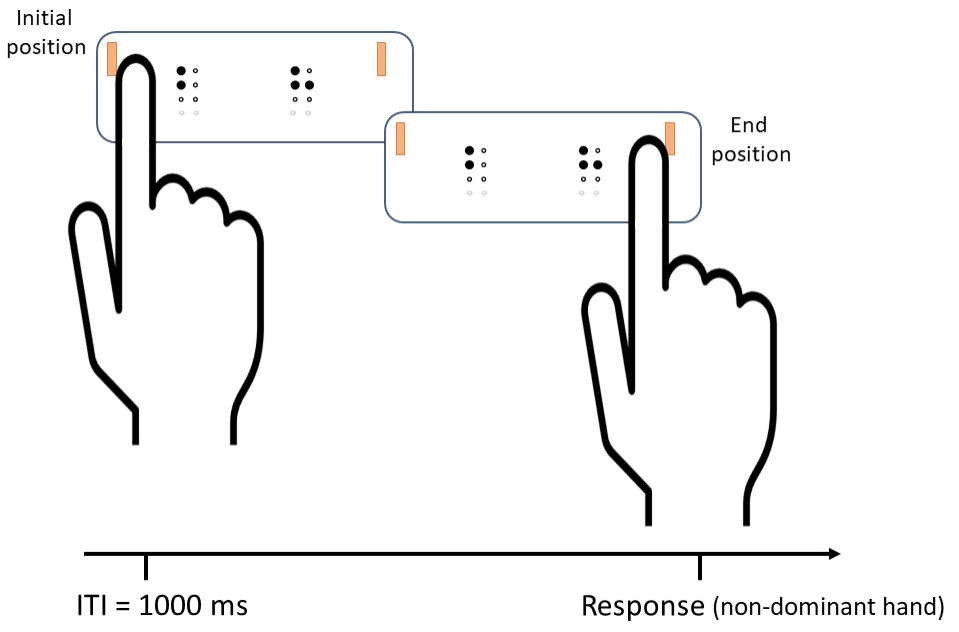
Diagram of a *different* trial in the same/different judgement task. Stimuli perception was done with the index finger of the dominant hand, and responses with the non-dominant hand. Inter-trial-interval (ITI) was one second—this allowed participants to reset the finger’s position. Every time a new trial appeared on display, the dots’ sound rising signaled participants to start the finger motion.

To touch the braille letters presented in the display, participants in the active procedure had to use the index finger of their dominant hand in a continuous left-to-right motion. Participants in the passive procedure placed their index fingertip on the start position and the braille display slided against it, moving for 5 cm at 38.9 mm/s. In both procedures, participants were instructed to use the middle and index fingers of the non-dominant hand to make responses by pressing the *same* and *different* keys (M and N, respectively) in the computer keyboard (see Figure 3).

After performing quality control checks (Gomez et al, 2017), we analyzed accuracy and latency data using (generalized) linear mixed-effects models in R (R Core Team, 2020) with the lme4 (Bates et al., 2015) and lmerTest packages (Kuznetsova et al., 2017). The fixed-effect factors were Procedure and Trial type; Subject was the random factor. For the accuracy data, we employed the binomial distribution (see Tables 1 and 2).

**Table 1:** Accuracy results from the linear mixed model.


VARIABLE	*b*	*SE*	*z*	*p*

Accuracy				

Procedure (active vs. passive)	0.065	0.069	0.940	0.347

Trial type (same vs. different)	–0.143	0.070	–2.057	0.040

Interaction (procedure*trial type)	–0.289	0.044	–6.531	< 0.001


*Note*: Overall, accuracy did not differ between the procedure groups. While accuracy was lower for different than for same trials, this difference was larger for the passive group than for the active group (14.0% vs. 8.1%, respectively).

**Table 2:** Latency results from the linear mixed model.


VARIABLE	*b*	*SE*	*t*	*p*

Response time				

Procedure (active vs. passive)	0.032	0.013	–7.648	< 0.001

Trial type (same vs. different)	–0.03	0.03	–1.141	0.254

Interaction (procedure*trial type)	0.01	0.02	0.772	0.440


*Note*: Overall, response times were longer for the active group than for the passive group. The other effects were not significant.

**Figure 4:**
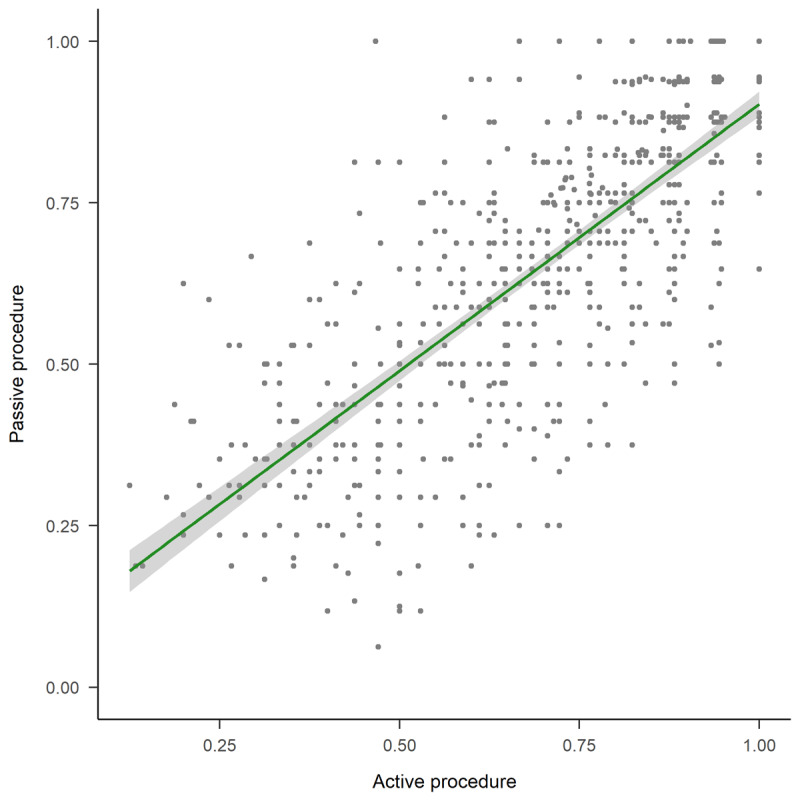
Scatterplot of the active and passive procedures. The x-axis shows the distribution of mean accuracy in the active condition and the y-axis shows the distribution of mean accuracy in the passive condition. Each data point represents a pair of letters, and the gray area represents the ±1 SE.

The Pearson correlation coefficient for the accuracy measures per trial between both procedures (i.e., active and passive) shows a strong linear association, *r* = 0.761 (see Figure 4). Critically, data collected using the dynamic (passive) version of TouchScope had less response time variability (SD_passive_ = 0.902s vs. SD_active_ = 1.563s) at no cost in overall accuracy.

## Discussion

The present article describes TouchScope, an easily reproducible system designed to control experiments on braille reading and tactile/haptic perception. To demonstrate its potential, we have shown that using the motorized mode of TouchScope in a same-different task does not alter participants’ accuracy and decreases the vast variability in response times relative to having participants actively move their fingers.

Furthermore, the static mode of TouchScope has been shown to be a useful tool to examine both tactile perception and braille reading. To examine context effects in the tactile modality, Baciero et al. (2021) developed a tactile version of the well-known Flanker effect (Eriksen & Eriksen, 1974) using TouchScope to present the stimuli and record participants’ responses. They simultaneously presented a target dot pattern (e.g., 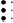) to participants’ middle fingerpad, and distractor patterns to the adjacent index and ring fingers (i.e., congruent or incongruent with the target; e.g., 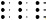, 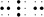). Participants had to identify the target stimulus by pressing the corresponding key on a computer keyboard. While there was a strong flanker effect, its temporal dynamics differ from those results found in the visual modality, thus suggesting the cognitive mechanisms that underlie this effect are guided by both modality-general and modality-specific processes. To examine braille orthographic processing, Baciero et al. (2022a, 2022b) tested skilled braille readers in two lexical decision studies. In the first one they created pseudowords by either transposing or replacing two adjacent letters of a word (e.g., aveinda vs. avearda, respectively. Baseword: avenida; avenue in Spanish). They found that transposed-letter pseudowords were classified less accurately and slower than replaced-letter pseudowords, showing that the noise associated with letter position coding in word recognition is not purely perceptual, and supporting the idea that serial order processing is a universal mechanism. In the second study (Baciero et al. 2022b), mispelled braille words were classified less accurately when the replaced letter was tactually similar to the original letter than when it was dissimilar^5^, thus revealing that the uncertainty during letter encoding in braille word recognition is not resolved as quickly as with common words in visual-word recognition (see Perea et al., 2022).

In sum, we have described the development of a tool that we believe offers researchers the possibility to design and conduct tactile research: 1) with reasonable control of the timing of presentation of the stimuli; 2) with a combination of behavioral and cognitive neuroscience techniques (e.g., recording electrophysiological measures); and 3) minimizing human errors related to the manual change of stimulus between trials. We believe that TouchScope will enable researchers to design novel techniques that measure response time to infer the cognitive mechanisms that underlie different tactile abilities. We hope TouchScope helps to expand tactile and haptic related research.

## Data Accessibility Statement

The code used to program TouchScope’s movement, the data recording, the stimuli, data files, and the R code for data wrangling and analyses regarding the experimental study are available at https://osf.io/tyzvp/.

## Additional File

The additional file for this article can be found as follows:

10.5334/joc.271.s1 Appendix. Computer code for Arduino IDE and bash shell.

## Footnotes

Note that non-automated techniques have also been used to study braille reading, for example with video recording the participants’ hands while reading braille (e.g., Millar, 1997), and manually giving participats objects to test tactile memory (e.g., Santaniello et al., 2018). However, those techniques lack the *automaticity* requirement because they require the manual coding of video frames, or physical events, potentially leading to human error and differences in results due to having different coders.

There is also free software for Windows: NVDA; although it is not built in the system. The version of the Mac OS in which the software’s device was implemented is Mojave.

We hope that future versions of the tool can be operated via a unique software that englobes the tasks now performed by Bash and Arduino in order to create a friendlier environment.

The maximum speed will depend on a combination of factors such as the type of stepper motor or the power supply, and these things could be modified, but for our purposes we tested it up to 200 rpm (120 mm/sec) and found reliable results.

Braille letter similarity was taken from the similarity matrices described in Baciero (2022), Section 4.1.
