# Supplementary material for: TouchScope: A Passive-Haptic Device to Investigate Tactile Perception Using a Refreshable Braille Display
Source: J Cogn. 2023 Apr 14;6(1):21. doi: 10.5334/joc.271 (PMC10162198; doi:10.5334/joc.271)
Supplement: Appendix. — Computer code for Arduino IDE and bash shell. [file joc-6-1-271-s1.pdf]

Appendix

Arduino IDE code:

```
// Install serial communication between bash shell and Arduino
IDE: brew.sh
// Install libraries for motor control (Adafruit Motor Shield) &
for serial communication ( advancedSerial) under "Sketch" in
toolbar
// Chech board ("Arduino Uno") and port (/dev/cu.usbmodem...)
under "Tools" in toolbar

#include <Wire.h>
#include <Adafruit_MotorShield.h>
#include "utility/Adafruit_MS_PWMServoDriver.h"
Adafruit_MotorShield AFMS = Adafruit_MotorShield();
Adafruit_StepperMotor *myMotor = AFMS.getStepper(200, 1);
void setup() {
  Serial.begin (9600); // Arduino ready to exchange messages with
Serial Monitor at 9600 bits/second (baud rate).
  pinMode (LED_BUILTIN, OUTPUT);
  AFMS.begin();  // create with the default frequency 1.6KHz
  digitalWrite(LED_BUILTIN, HIGH);
}
void loop() {
  int movement = Serial.read(); // Read the information in the
serial monitor. Via the Bash script we send a “1” for “forward”
movement (right to left)and a “2” for “backward” movement (left
to right)
  if (movement == '1') {
    myMotor->setSpeed(7000); //Here you set the speed in rpm
    myMotor -> step(200, FORWARD, DOUBLE); // Here you set the
number of steps
      }
  else if (movement =='2') {
    myMotor->setSpeed(65); // 65rpm
    myMotor -> step(200, BACKWARD, DOUBLE); //200 steps
```

```
  }
}
```

Bash Shell script (no movement):

```
# setup a new data file (e.g., one per participant)
echo “data file name (no spaces)”
read datafile;
# Exp Variables
echo “enter ITI in seconds” #inter-trial interval
read ITI;
echo "enter stimuli file name" #stimuli list
read path;

echo "     ";
sleep $ITI;

# Send tidy data to the datafile”>” creates the file. The column
name is the following line between quotations
echo "IT1 IT2 KEY RT" > "$datafile"
IFS=$'\n'  #new line

for p in `cat "$path"`; #loop
do

ITEM="$p"; #show trial
clear;
start=$(gdate +%s.%N); #timer
read -n 1 -s -r -p "$ITEM" KEY; #read key pressed
RT=$(echo "$(gdate +%s.%N) - $start" | bc); #response time
printf  "$ITEM  $KEY %.3f\n" $RT  >> "$datafile"; #save
clear;
sleep $ITI; #wait inter-trial interval

done
```

Bash Shell script (with movement):

```
# setup a new data file (e.g., one per participant)
echo “data file name (no spaces)”
read datafile;
# Exp Variables
echo “enter ITI in seconds” #inter-trial interval
read ITI;
echo "enter stimuli file name" #stimuli list
read path;
```

```
echo "     ";
sleep $ITI;

# Send tidy data to the datafile”>” creates the file. The column
name is the following line between quotations
echo "IT1 IT2 KEY RT" > "$datafile"
IFS=$'\n'  #new line

for p in `cat "$path"`; #loop
do

ITEM="$p"; #show trial
./arduino-serial -b 9600 -p /dev/cu.usbmodem1411 -s 2 %>
/dev/null; #send a “2” to Arduino Serial Monitor
clear;
start=$(gdate +%s.%N); #timer
read -n 1 -s -r -p "$ITEM" KEY; #key pressed
RT=$(echo "$(gdate +%s.%N) - $start" | bc); #response time
printf  "$ITEM  $KEY %.3f\n" $RT  >> "$datafile"; #save data
clear;
./arduino-serial -b 9600 -p /dev/cu.usbmodem1411 -s 1 %>
/dev/null; #send a “1” to Arduino Serial Monitor
sleep $ITI; #wait inter-trial interval

done
```
